# Novel Core–Shell Metal Oxide Nanofibers with Advanced Optical and Magnetic Properties Deposited by Co-Axial Electrospinning

**DOI:** 10.3390/nano15131026

**Published:** 2025-07-02

**Authors:** Roman Viter, Viktor Zabolotnii, Martin Sahul, Mária Čaplovičová, Iryna Tepliakova, Viesturs Sints, Ambra Fioravanti

**Affiliations:** 1 Faculty of Science and Technology, University of Latvia, 19 Raina Blvd, LV 1586 Riga, Latvia; viktor.zabolotnii@lu.lv (V.Z.); iryna.tepliakova@lu.lv (I.T.); viesturs.sints@lu.lv (V.S.); 2 Institute of Materials Science, Slovak University of Technology in Bratislava, Jána Bottu 25, 917 24 Trnava, Slovakia; martin.sahul@stuba.sk; 3 Centre for Nanodiagnostics of Materials, Slovak University of Technology in Bratislava, Vazovova 5, 812 43 Bratislava, Slovakia; maria.caplovicova@stuba.sk; 4 Institute of Sciences and Technologies for Sustainable Energy and Mobility (STEMS), National Research Council (CNR), Via Canal Bianco, 28, 44124 Ferrara, Italy; ambra.fioravanti@stems.cnr.it

**Keywords:** core–shell nanofibers, co-axial electrospinning, phase transitions, visible-light absorption, magnetic properties

## Abstract

Co-axial electrospinning is one of the facile methods for the fabrication of core–shell metal oxides for environmental applications. Indeed, core–shell architectures featuring a magnetic core and a photocatalytic shell represent a novel approach to catalytic nanostructures in applications such as water treatment and pollutant removal via magnetic separation. This study focuses on the fabrication of novel Fe_3_O_4_-Fe_2_NiO_4_/NiO core–shell nanofibers with enhanced optical and magnetic properties using co-axial electrospinning. The aim is to optimize the fabrication parameters, particularly the amount of metal precursor in the starting solutions, to achieve well-defined core and shell structures (rather than single-phase spinels), and to investigate phase transitions, structural characteristics, as well as the optical and magnetic properties of the resulting nanofibers. Raman, XRD, and XPS results show several phases and high defect concentration in the NiO shell. The Fe_3_O_4_-Fe_2_NiO_4_/NiO core–shell nanofibers exhibit strong visible-light absorption and significant magnetization. These advanced properties highlight their potential in photocatalytic applications.

## 1. Introduction

Co-axial electrospinning is a powerful, versatile, and low-cost technique for the development of core–shell nanostructures with tailored properties. It has been widely used to fabricate core–shell polymeric nanofibers for wound healing, drug delivery, and filtration [1,2,3]. More recently, co-axial electrospinning has been applied to prepare functional nanomaterials, including oxide/oxide metal/oxide, metal/metal nanofibers, and hollow metal oxide nanotubes [4,5,6,7].

Metal oxide–metal oxide heterostructure plays an essential role in the applications of nanomaterials in sensors, electrocatalysis, and photocatalysis [8,9]. Metal oxide–metal oxide heterostructures offer several advantages, including increased surface area, enhanced redox activity, stable interfaces, excellent electrical conductivity, improved ion diffusion kinetics, high specific capacitance, good cycling stability, and overall enhanced electrical and optical properties resulting from interface defects and improved charge separation [8,9]. The integration of optical and magnetic properties in core–shell nanofibers opens new possibilities for the development of low-cost photocatalytic and photoelectrocatalytic systems with magnetic separability. Typical sol–gel fabrication of magnetic core–shell nanostructures assumes the use of magnetic iron oxides in the core and other functional oxides (like TiO_2_, NiO, WO_3_, MoO_2_, etc.) in the shell. Due to the interface formed into the core–shell heterostructure, efficient charge separation takes place, enhancing photocatalytic and electrocatalytic properties [10,11].

It was reported that core–shell metal oxide nanofibers showed a high surface-to-volume ratio, stable interface, and good functional properties. For instance, ZnO/In_2_O_3_, SnO_2_/In_2_O_3_, and TiO_2_ were used for gas sensors and catalysis applications [8,9]. A successful approach for magnetic metal/carbon core–shell nanofibers was reported [12]. At the same time, no reports on the fabrication of magnetic core–shell metal oxide nanofibers were reported. The potential applications do not involve other properties of metal oxides, such as optical absorption, catalytic properties, magnetic properties, etc. The main achievements and applications of core–shell nanofibers are presented in Table 1.

The fabrication of magnetic core–shell nanofibers could depend on the ratio between precursors in the core and the shell, as well as the temperature of annealing. Iron oxides are typical materials used for magnetic cores. It is known that iron tends to form non-magnetic spinels with other oxides at high temperature treatment (over 500 °C). Interdiffusion and spinel forming might change magnetic, optical, and catalytic properties of the core–shell nanostructures [22]. This phenomenon is not well-discovered for co-axial electrospinning of magnetic metal oxide nanostructures.

Recently, we reported the fabrication of core–shell ZnFe_2_O_4_/ZnO nanofibers by co-axial electrospinning. It was found that the obtained samples showed no magnetic properties. Due to spinel forming, the core of the fiber showed 120 nm ZnFe_2_O_4_, whereas the ZnO shell was formed by ZnO particles with an average size of 20–30 nm. The samples showed good photoelectrochemical properties. However, the primary goal of achieving magnetic metal oxide/metal oxide nanofibers was not released.

Transition metals and their oxides (NiO, MoO_2_, MnO_2_, Co_3_O_4_) are promising materials for sensor, photoelectrocatalytic, and photocatalytic applications [23,24]. Due to a number of oxidation states, they show enhanced interaction with target molecules and higher performance. Recent reports show successful fabrication of Ni/C, Fe_3_O_4_/C, and Ni-Co spinel nanofibers by the co-axial electrospinning method [12,23,24,25,26]. However, no reports on the fabrication of magnetic metal oxide/metal oxide nanofibers, design, and main properties of such nanostructures have been published yet.

The analysis of the literature shows that iron oxide has not been studied as a potential core material for magnetic core–shell nanofibers. Core–shell composition of iron oxide and other transition oxides in electrospun nanofibers has not been studied in detail. The role of core and shell precursors, structural, optical, and magnetic properties of the formed nanofibers have not been investigated yet. Possible phase transitions in core–shell metal oxide nanofibers have been partially studied. Therefore, processing of opto-magnetic metal oxide core–shell nanofibers with tailored structure, optical, and magnetic properties is an actual topic for potential photocatalysis and photoelectrochemical applications.

Chemical properties of polymers, used in the core and shell, are key parameters in the co-axial electrospinning [27]. Different polymers have been used for the fabrication of core–shell nanofibers and hollow nanotubes by co-axial electrospinning [27]. The most commonly used polymers were Polyvinylpyrrolidone (PVP) [14,15,16], Polysterene (PS) [28], poly(D,L-lactide) (PLA) [29], and Polyacrylonitrile (PAN) [30]. The recent reports showed the common use of PVP/PVP as core and shell polymers for the fabrication of core–shell metal oxide nanofibers [14,15,16]. However, the promising approach in the fabrication of hollow TiO_2_ nanotubes demonstrated using PAN/PVP as core and shell polymers, respectively [30]. The main requirement for the core and shell polymers is to avoid the formation of non-spinnable gels on the needle tip during their altering.

In the present paper, we report on the fabrication and optimization of opto-magnetic core–shell metal oxide nanofibers by co-axial electrospinning. The formation of the core–shell fibers with iron oxide in the core and Mn, Mo, Co, and Ni oxides in the shell has been investigated. The role of core and shell polymers and precursors in forming the structure, optical, and magnetic properties of the core–shell nanofibers was discussed. Phase transitions, spinel forming, and optimal parameters for the fabrication of core–shell nanofibers with tunable properties have been analyzed.

In the present paper, we report on the fabrication and optimization of opto-magnetic core–shell metal oxide nanofibers by co-axial electrospinning. Forming of the core–shell fibers with iron oxide in the core and Mn, Mo, Co, and Ni oxides in the shell has been investigated. The role of core and shell precursors in forming the structure, optical, and magnetic properties of the core–shell nanofibers was discussed. Phase transitions, spinel forming, and optimal parameters for the fabrication of core–shell nanofibers with tunable properties have been analyzed.

## 2. Experimental

### 2.1. Materials

Polyvinylpyrrolidone (PVP) (Mw 1,300,000), Polyacrylonitrile (PAN), N, N-Dimethylformamide (DMF), Fe(NO_3_)_2_ 9H_2_O, nickel acetate (NiAc), Co(NO_3_)_2_ 6H_2_O, Mn(NO_3_)_2_ 6H_2_O, and (NH_4_)_6_Mo_7_O_24_ 4H_2_O were purchased from Sigma Aldrich, St. Louis, MO, USA.

### 2.2. Solution Fabrication

The core solution (Solution A) was prepared as follows: DMF (4 mL) was heated up to 75 °C. PAN (0.41 g) was added to hot DMF and stirred (Solution 1). DMF (1 mL) and Fe(NO_3_)_2_ (0.3–0.6 g) were mixed and ultrasonicated for 15 min (Solution 2). When PAN in Solution 1 was fully dissolved, Solution 2 was added to Solution 1 under continuous stirring. The fabricated Solution A was stirred overnight at room temperature.

The shell solution (Solution B) was prepared as follows: DMF (3 mL) was heated up to 50 °C. PVP (0.65 g) was added to hot DMF and stirred (Solution 3). DMF (2 mL) and metal salts (NiAc (0.15–0.5 g), Co(NO_3_)_2_ 6H_2_O (0.5 g), Mn(NO_3_)_2_ 6H_2_O (0.5 g), (NH_4_)_6_Mo_7_O_24_ 4H_2_O (0.5 g)) were mixed and ultrasonicated for 30 min (Solution 4). When PVP in Solution 3 was fully dissolved, Solution 4 was added to Solution 3 under continuous stirring. The fabricated Solution B was stirred overnight at room temperature.

The parameters of Solutions A and B were recalculated using the weight fractions (w/w_solution_) of polymers and metal precursors. We obtained the following weight concentrations of the polymer and iron precursor in the tested Solution A: PAN 7.55% Fe 5.5% (PAN 0.41 g and 0.3 g iron nitrate); PAN 7.41% Fe 7.23% (PAN 0.41 g and 0.4 g iron nitrate); PAN 7.28% Fe 8.89% (PAN 0.41 g and 0.5 g iron nitrate); and PAN 7.15% Fe 10.4% (PAN 0.41 g and 0.6 g iron nitrate).

The following weight concentrations of the PVP and metal precursor were obtained in the tested Solution B: PVP 11.7% Me (metal) 2.27% (0.65 g PVP precursor and 0.15 g Me precursor); PVP 11.4% Ni 5.29% (0.65 g PVP precursor and 0.3 g Me precursor); and PVP 11.1% Me 8.51% (0.65 g PVP precursor and 0.5 g Me precursor).

### 2.3. Electrospinning

Solutions A and B were loaded in separate 5 mL plastic syringes and attached to a co-axial needle (Linari Engineering, Pisa, Italy) with plastic tubes. Co-axial needle has an inner diameter of 0.5 mm and an outer diameter of 1 mm. The syringes were installed in two independent syringe pumps and set up with pump rates of 300 μL/h (Solution A) and 400 μL/h (Solution B). The needle was installed into the spinning camera 20 cm above the collector. The collector was covered by an aluminum foil. The collector’s rotating speed was 200 rpm. The voltage applied between the needle and the collector was 20 kV.

Control samples of NiO nanotubes and iron oxide nanofibers were deposited without metal precursors in the core and shell, respectively.

As-prepared nanofibers were dried in a vacuum overnight at room temperature and annealed at 500 °C for 1 h.

Polymers, used in the core and shell solutions, are key parameters in co-axial electrospinning. In the present work, different polymers were tested in core and shell solutions, such as PVP/PVP, Polycaprolactone (PCL)/PVP, PAN/PCL [14,15,16]. Our results showed forming of gels on the tip of the co-axial needle or spinel forming after annealing. Our findings showed sufficient use of PAN/PVP as core and shell polymers, respectively, similarly to results reported in [30].

### 2.4. Characterization

X-ray diffraction (XRD) measurements for analysis of phases in the core–shell nanofibers were performed using an Empyrean X-ray diffractometer (Malvern Panalytical, Malvern, UK) equipped with a cobalt (Cu) radiation source (λ = 1.5406 Å). The instrument was operated at an accelerating voltage of 40 kV and a current of 30 mA. The incident beam optics included a programmable divergence slit (1/32°), an anti-scatter slit (1/16°), a Soller slit (0.04 rad), and a fixed mask with a 10 mm aperture. On the diffracted beam side, the configuration comprised a β-filter, Soller slits (0.04 rad), a programmable anti-scatter slit (1/16°), and a PIXcel3D detector (Medipix3) (Malvern Panalytical, Malvern, UK) operated in scanning line mode. This setup ensured high-resolution data collection for precise phase identification and structural analysis.

To analyze the molecular vibrations and bonds in the obtained materials, Raman spectroscopy measurements were performed in a WiTeC Alpha 300R microRaman system (WITec Wissenschaftliche Instrumente und Technologie GmbH, Ulm, Germany) equipped with a laser (532 nm excitation wavelength).

X-ray Photoelectron Spectroscopy (XPS) was used for oxidation state evaluation and chemical shift determination using a Thermo Fisher Scientific Escalab Xi+ spectrometer (Thermo Fisher Scientific, Waltham, MA, USA) in high vacuum conditions. The tested core–shell nanofibers were deposited on carbon pads, and any loose material was removed with high-velocity airflow. Charge compensation (flood gun, standard mode) and surface etching (ion gun, mild conditions for 10 s) were used to remove surface contamination and reduce surface charging. XPS data were analyzed using Avantage 5.2995 software, and the advantageous carbon peak at 284.8 eV was used as a calibration point.

The morphology and size of the core–shell nanofibers were examined using a high-resolution field emission scanning electron microscope (FE-SEM, JEOL JSM-7600F, JEOL, Tokyo, Japan). Imaging was conducted at an accelerating voltage of 5 keV. Both secondary electron imaging (SEI) and low-angle backscattered electron (LABE) modes were employed to provide complementary information on surface topography and material contrast. Before imaging, the core–shell nanofibers were mounted on conductive carbon tape and gently cleaned with compressed nitrogen to remove loosely attached fibers.

High-resolution transmission electron microscopy (HR-TEM) was carried out using a double aberration-corrected JEM-ARM200CF microscope (JEOL, Tokyo, Japan), operated at an accelerating voltage of 200 kV. The core–shell nanofibers were dispersed in ethanol and sonicated for 10 min for TEM sample preparation. A drop of the resulting suspension was deposited onto a copper TEM grid coated with a holey carbon film, followed by air drying. Energy-dispersive X-ray spectroscopy (EDX) was conducted using the same TEM instrument, which was equipped with a large-angle JEOL JED-2300T CENTURIO silicon drift detector (SDD) featuring a solid angle of 0.98 steradians and a detection area of 100 mm^2^. This analysis enabled the identification and spatial distribution of dopant elements within the samples.

Optical properties of the core–shell nanofibers were studied by diffuse reflectance spectroscopy in the UV–Visible range, Ocean Optics fiber optic light source (DH2000, 250–900 nm, Orlando, FL, USA), integrating sphere (Ocean Optics, IS-8, Orlando, FL, USA), and fiber optic spectrometer (Ocean Optics HR4000, Orlando, FL, USA).

Magnetic properties of the core–shell nanofibers were characterized by vibrating sample magnetometry (VSM). Measurements were performed with a Lake Shore Cryotronics Co., Westerville, OH, USA, model 7404 VSM vibrational sample magnetometer. The range of the magnetizing field was −1 T to 1 T, applied in a sequence starting with zero field and then sweeping through the range twice, so that both zero field susceptibility and any possible hysteresis can be recorded. The sample holders were found to have non-negligible magnetic properties (especially in the case of less magnetic samples). Therefore, the magnetization of each holder was measured prior to sample measurements and subtracted from the results.

## 3. Results

### 3.1. Role of the Shell Precursor to Fabrication of the Core–Shell Nanofibers

**Figure 1:**
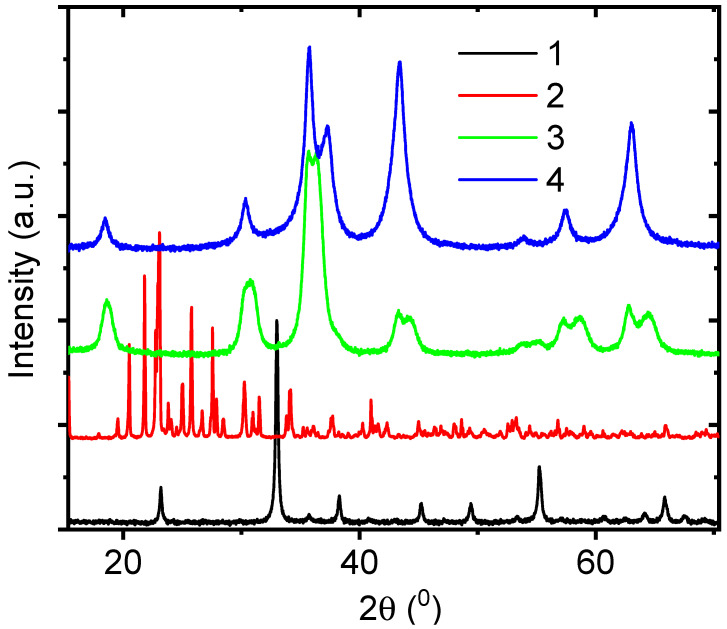
XRD spectra of core–shell nanostructures: 1—FeMn, 2—FeMo, 3—FeCo, 4—FeNi.

The samples, deposited from solutions with Fe nitrate in the core and Mn, Mo, Co, and Ni precursors in the shell, are named FeMn, FeMo, FeCo, and FeNi, respectively. XRD spectra of the fabricated core–shell nanofibers are shown in Figure 1. Analysis of the peak positions showed no core–shell structure forming for FeMn, FeMo, and FeCo samples [31,32,33].

For instance, the peaks located at 23.15, 32.97, 35.8, 38.24, 45.2, 49.37, and 55.23 2θ (Figure 1, curve 1) corresponded to FeMnO_3_ (JCPDS Card No. 76-0076) [31]. The XRD peaks observed in Figure 1, curve 2, located at 19.53, 20.47, 21.78, 23.15, 23.8, 25.0, 25.82, 26.7, 27.56, and 30.3 2θ, corresponded to Fe_2_(MoO_4_)_3_ (JCPDS Card No. 85-2287) [32,33]. The XRD peaks observed in Figure 1, curve 3, located at 2θ 36.29, 44.39, 53.8, 58.63, 62.8, and 64.48, were assigned to FeCo_2_O_4_ [34,35,36]. (JCPDS Card No. 22-1086).

The broad XRD peaks observed in Figure 1, curve 3, located at 18.6, 30.66, 35.72, 55.2, 43.3, and 57.18 2θ, could correspond to (111), (220), (311), (400), (422), and (511) crystalline lattice planes of FeCo_2_O_4_ (JCPDS Card No. 22-1086) and Fe_3_O_4_ (JCPDS card number 11-0614) [37].

XRD analysis of FeNi samples (Figure 1, curve 4) showed peaks at 2θ 18.6, 30.23, 35.8, 53.85, and 57.53 corresponding to Fe_3_O_4_ (JCPDS card number 11-0614) peaks at 2θ 37.25, 43.38, and 63.04 corresponding to NiO phase (JCPDS card number 47-1049) [25]. The XRD analysis shows that core–shell structures are possibly formed for FeNi samples.

From XRD data analysis, we conclude that high temperature annealing results in an interdiffusion of metal atoms between the Fe-based core and the Mn- and Mo-based shells. The FeCo samples, showing a possible core–shell structure, were excluded from further analysis as SEM studies did not show the formation of nanofibers (Figure S1).

Core–shell nanofibers, formed by the combination of Fe-based precursor in the core and Ni-based precursor in the shell, initially showed two separate metal oxide phases. Optimization of the fabrication parameters should be investigated.

### 3.2. Optimization of Variation of Core Concentration in FeNi Core–Shell Nanofibers

The optimization of the core–shell fabrication was performed by keeping the concentration of polymeric solutions, their pumping speeds, applied voltage, and the distance between the needle and the collector constant.

#### 3.2.1. Variation of the Fe Nitrate Concentration in the Core Solution

The first optimization was addressed to study the effects of the core composition at fixed shell parameters. The deposited samples had the following parameters:

- **FeNi35**: PAN 7.55% Fe 5.5% (0.3 g Fe nitrate)/PVP 11.1% Ni 8.51% (0.5 g Ni acetate);
- **FeNi45**: PAN 7.41% Fe 7.23% (0.4 g Fe nitrate)/PVP 11.1% Ni 8.51% (0.5 g Ni acetate);
- **FeNi55**: PAN 7.28% Fe 8.89% (0.5 g Fe nitrate)/PVP 11.1% Ni 8.51% (0.5 g Ni acetate);
- **FeNi65**: PAN 7.15% Fe 10.4% (0.6 g Fe nitrate)/PVP 11.1% Ni 8.51% (0.5 g Ni acetate).

Control samples of iron core/empty shell and empty core/nickel shell were assigned as FeNF and NiNT, respectively.

**Figure 2:**
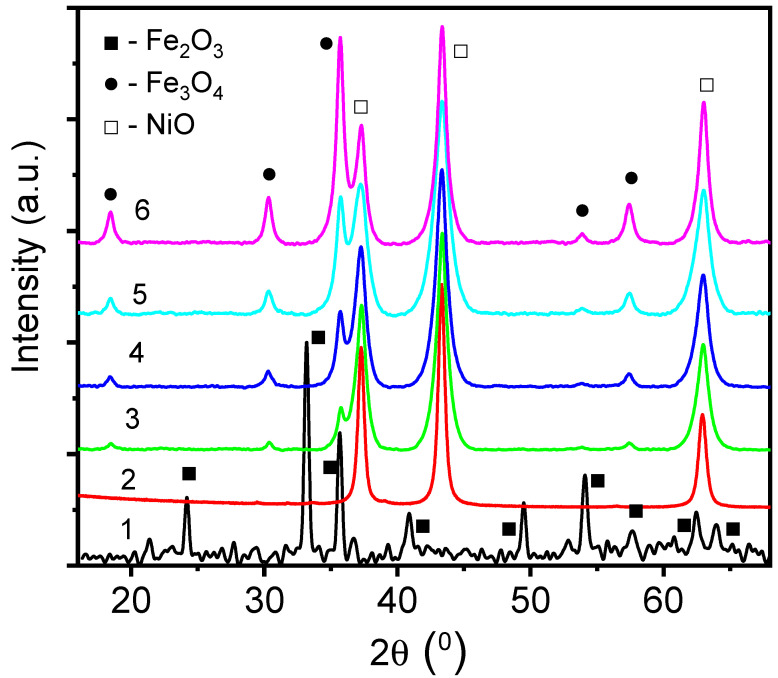
XRD of core–shell nanofibers and control samples: 1—FeNF, 2—NiNT, 3—FeNi35, 4—FeNi45, 5—FeNi55, 6—FeNi65.

XRD spectra of the core–shell nanofibers are shown in Figure 2. Analysis of the XRD spectra showed that Fe_2_O_3_ is formed in the case of the control FeNF nanofibers (Figure 2, curve 1), whereas Fe_3_O_4_ is formed in core–shell nanofibers (Figure 2, curves 3–6). The phase of NiO has been identified in NiNT and FeNi nanostructures (Figure 2, curves 2–6). The full width of the half maximum (FWHM) of NiO XRD peaks slightly increased for core–shell FeNi nanostructures compared to NiNT. As no correlation between [Fe/Ni] concentrations and the FWHM was found, we assume that the peak widening could be explained by the lattice strain at the interface of the core–shell structure.

Analysis of the XRD peaks of core–shell nanofibers showed the increase in the Fe_3_O_4_ peaks’ intensity with the increase in the [Fe/Ni] concentration ratio, indicating the increase in the core.

**Figure 3:**
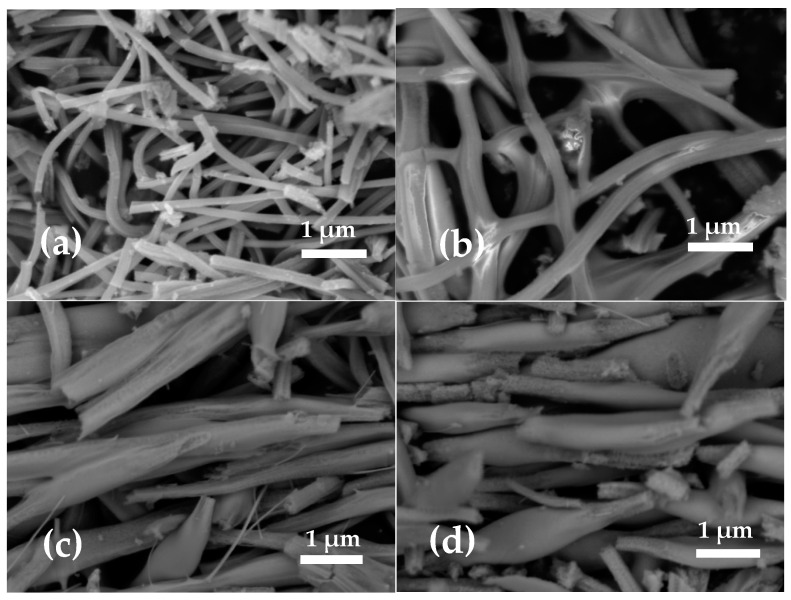
SEM images of core–shell nanostructures: (**a**) FeNi35, (**b**) FeNi45, (**c**) FeNi55, (**d**) FeNi65.

SEM images of the FeNi35, FeNi45, FeNi55, and FeNi65 are presented in Figure 3. The SEM images show that the core precursor concentration is a crucial parameter for the fabrication of homogeneous and well-shaped nanofibers. The nanofibers, fabricated from core solutions with iron nitrate concentration more than 5.5%, showed bead-like structures. We assume that the observed results could be explained by the drastic increase in the conductivity and viscosity of the core [14,26,27]. It was shown that the conductivity effect results in a decrease in the diameter of the produced fibers [14].

As was shown previously, within the variation of core parameters, the PAN concentration reduced from 7.55% (*w*/*w*) to 7.15% (*w*/*w*), whereas iron nitrate concentration increased from 5.5% (*w*/*w*) to 10.4% (*w*/*w*). The measured viscosity of the core solutions changed to 5% from 0.7 mPa to 0.73 mPa with changes in the polymer and precursor concentrations. We suppose that the increase in the iron nitrate concentration in the core solution made the conductivity of the core solution dominate in the spinning process. The increased conductivity of the core solution results in damage to the jet flow and bead-like shapes of the fibers [30,38,39,40]. As SEM does not show well-shaped nanofibers, we need to reduce the iron nitrate concentration in the core solution to 5.5% (*w*/*w*) core and keep it fixed. Nickel acetate concentration (*w*/*w*) in the shell solution will be changed from 2.27% to 8.51%.

#### 3.2.2. Variation of the Ni Acetate Concentration in the Shell Solution

The second optimization was addressed to study the effects of the shell composition at fixed core parameters. The deposited samples had the following parameters:

- **FeNi31**: PAN 7.55% Fe 5.5% (0.3 g Fe nitrate)/PVP 11.7% Ni 2.27% (0.15 g Ni acetate);
- **FeNi33**: PAN 7.55% Fe 5.5% (0.3 g Fe nitrate)/PVP 11.4% Ni 5.29% (0.3 g Ni acetate);
- **FeNi35**: PAN 7.55% Fe 5.5% (0.3 g Fe nitrate)/PVP 11.1% Ni 8.51% (0.5 g Ni acetate).

Control samples of iron core/empty shell and empty core/nickel shell were assigned as FeNF and NiNT, respectively.

**Figure 4:**
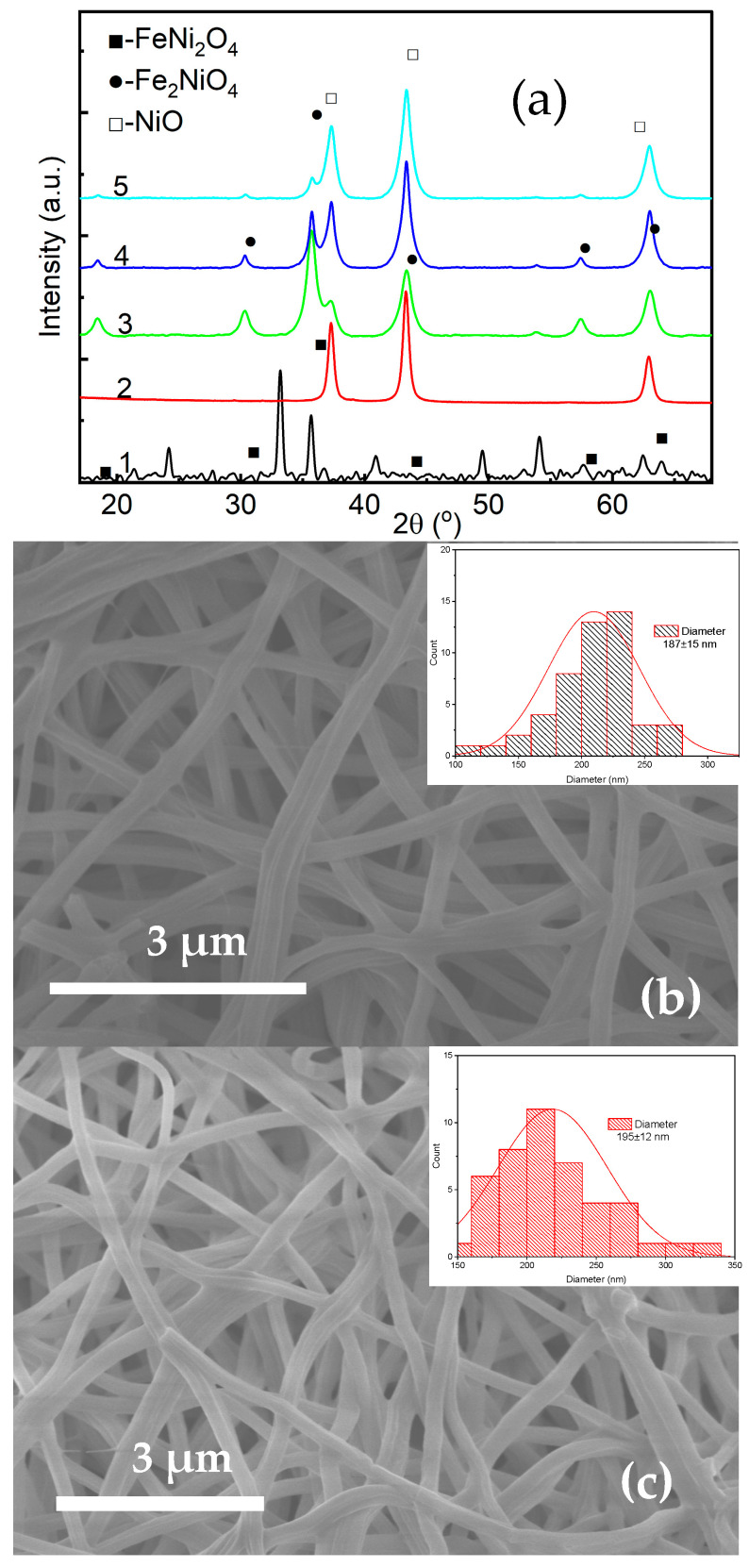

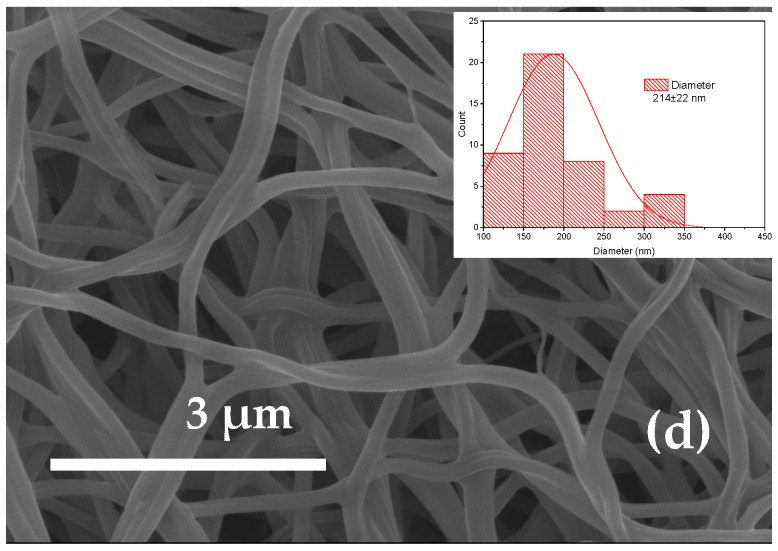
Structure properties of the core–shell nanofibers, measured by XRD and SEM: (**a**) XRD spectra of optimized FeNi: 1—FeNF, 2—NiNT, 3—FeNi31, 4—FeNi33, 5—FeNi35, (**b**) SEM and diameter distribution of FeNi31, (**c**) SEM and diameter distribution of FeNi33, (**d**) SEM and diameter distribution of FeNi35.

Figure 4a presents X-ray diffraction patterns of the synthesized core–shell nanofibers with varying shell parameters. XRD spectra indicate the presence of Fe_3_O_4_ (magnetite) and NiO (nickel oxide) as dominant crystalline phases. The peak intensity of the Fe_3_O_4_ and NiO depended on the [Fe/Ni] concentration ratio. The increase in the [Fe/Ni] led to an increase in the NiO XRD peaks, indicating an increase in the shell dimensions.

Figure 4b–d show SEM images of the core–shell nanofibers with size distributions of the fiber diameters. The fiber diameters, determined from SEM images, show the following values: 187 ± 15 nm, 195 ± 12 nm, and 214 ± 22 nm for FeNi31, FeNi33, and FeNi35 nanostructures, respectively. The average length of the nanofibers was 7 μm.

### 3.3. Structure, Optical, and Magnetic Properties of the Fe_3_O_4_ and NiO Core–Shell Nanofibers

**Figure 5:**
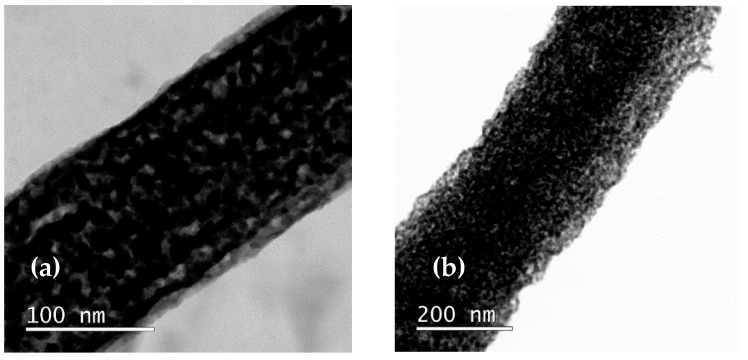

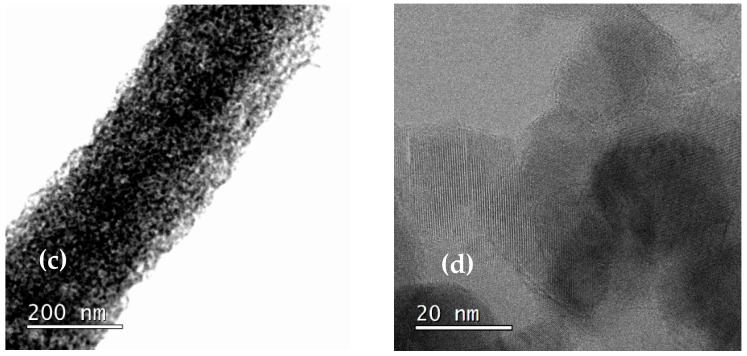
Structural properties of the core–shell nanofibers, measured by TEM: (**a**) FeNi31, (**b**) FeNi33, (**c**) FeNi35, (**d**) TEM of core–shell fiber surface indicating NiO phase.

It was found that the change in the shell concentration affected the structure parameters of the nanofibers. The TEM image of the shell is shown in Figure 5. The linear dimensions of the core and shell are summarized in Table 2.

The interplane distance, determined from TEM of the surface area (Figure 5d) (d = 0.2433 ± 0.0056 nm), showed plane (110) of NiO with lattice constant a = 0.4215 ± 0.0097 nm.

The obtained lattice constant showed an increase in value compared to the nanocrystalline NiO. This could point to the formation of structural defects in NiO.

**Figure 6:**
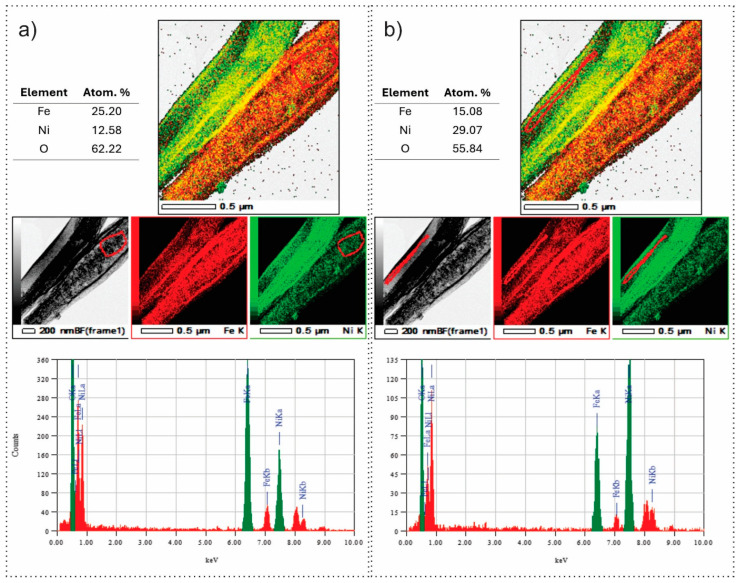
TEM-EDS maps of Fe, Ni, and O; EDS elemental composition and spectra taken from the area of (**a**) core and (**b**) shell of the sample FeNi35 (red circles show area of selection).

Figure 6 presents TEM-EDS elemental mapping and compositional analysis of a FeNi 35 nanowire sample, highlighting distinct differences between the two regions associated with a proposed core–shell structure. Area on Figure 6a corresponds to the region interpreted as the core, where EDS mapping reveals a higher concentration of iron relative to nickel, with atomic percentages of Fe at 25.20%, Ni at 12.58%, and O at 62.22%. The composite and individual elemental maps show a relatively uniform distribution of oxygen. At the same time, Fe is more abundant than Ni, consistent with a Fe:Ni ratio of approximately 2:1. This stoichiometry closely matches that of Fe_2_NiO_4_, supporting the identification of this region as the outer shell of the nanowire. In contrast, the area of Figure 6b corresponds to the nanowire shell, where the EDS data indicate a reversed elemental ratio with Fe at 15.08%, Ni at 29.07%, and O at 55.84%. The Ni-rich composition aligns well with the stoichiometry of FeNi_2_O_4_, suggesting that this region forms the core of the nanowire. In both regions, oxygen is homogeneously distributed, indicating complete oxidation. The EDS spectra further confirm these trends, with the area of Figure 6a showing a dominant Fe signal and the area of Figure 6b showing a dominant Ni signal, correlating well with the corresponding atomic concentrations. The mapping, quantitative data, and spectral profiles provide compelling evidence of a compositional gradient consistent with a FeNi-based core–shell nanostructure.

Closer inspection, supported by TEM-EDS data (Figure 6), revealed additional contributions from iron nickel oxide phases, specifically FeNi_2_O_4_ (ICSD 01-074-6507) and Fe_2_NiO_4_ (ICSD 01-071-3850). These mixed oxide phases have similar crystallographic parameters and space groups (e.g., Fd-3m for FeNi_2_O_4_), leading to overlapping diffraction peaks with those of Fe_3_O_4_ and NiO, complicating straightforward identification in the XRD patterns.

These spinel-type mixed oxides align with expected interdiffusion and solid-state reactions between the iron and nickel components during the thermal processing of the core–shell nanofibers. This data will be compared by analysis of Raman, XPS, and optical methods.

**Figure 7:**
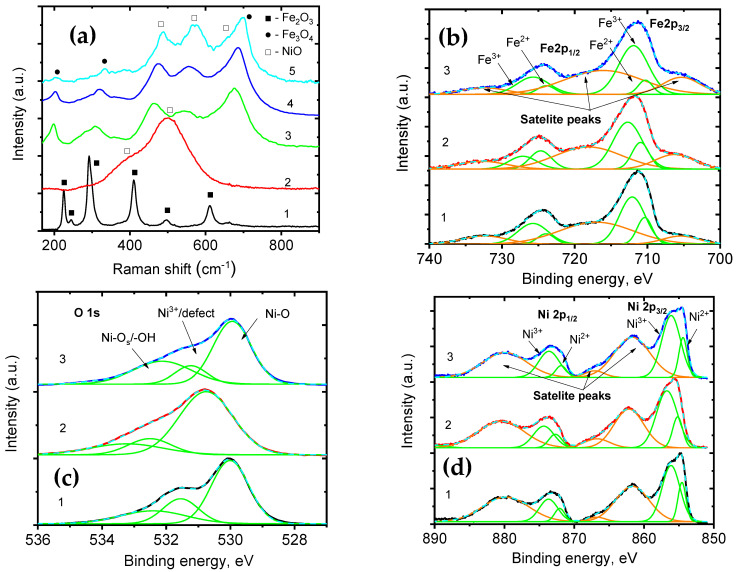
Structure properties of the core–shell nanofibers, measured by Raman and XPS: (**a**) Raman spectra: 1—FeNF, 2—NiNT, 3—FeNi31, 4—FeNi33, 5—FeNi35; (**b**) XPS of Fe2p: 1—FeNi31, 2—FeNi33, 3—FeNi35; FeNi31; (**c**) XPS of Ni2p: 1—FeNi31, 2—FeNi33, 3—FeNi35; FeNi31; (**d**) XPS of O 1S: 1—FeNi31, 2—FeNi33, 3—FeNi35.

Figure 7a shows Raman spectra of core–shell nanofibers, NiO nanotubes, and iron oxide nanofibers. Raman peaks, identified at 223, 291, 409, 496, and 611 cm^−1^ (Figure 7a, curve 1), correspond to A_g1_, E_g1_, E_g1_, A_g1_, and E_g1_ vibrational modes of the Fe_2_O_3_ [41]. The Raman spectrum of NiO nanotubes (Figure 7a, curve 2) showed peaks, located at 401 cm^−1^ (1P-TO), 499 cm^−1^ (1P-M), and 599 cm^−1^ (1-P LO), confirming NiO phase [42].

The Raman spectra of core–shell nanofibers (Figure 7a, curves 3–5) showed a significant difference from the Raman peaks, related to pristine Fe_2_O_3_ and NiO. The peaks, exhibited at 198 (T_2g_(1)), 328 (E_g_), and 698 cm^−1^ (A_g1_), could correspond to the Fe_3_O_4_ phase [43,44], being in good agreement with XRD measurements [19,45]. The Raman peaks, assigned at 465 (1P-TO), 544 (1P + 1M), and 659 cm^−1^ (1-P LO), could correspond to NiO [42,46,47].

Raman spectra of the FeNi31–35 should correlate with TEM/EDS analysis. According to TEM/EDS analysis of the samples, two spinel phases, NiFe_2_O_4_ and FeNi_2_O_4,_ could be formed. In the Raman spectra (Figure 7, curves 3–5), we explain Raman peaks at 460–480 cm^−1^ and 560–580 cm^−1^ with Ni-O vibrations in NiO, NiFe_2_O_4,_ and FeNi_2_O_4_ crystalline lattices [48,49,50,51,52,53]. The peak at 675–692 cm^−1^ is associated with Fe-O vibrations in Fe_3_O_4_, NiFe_2_O_4,_ and FeNi_2_O_4_ crystalline lattices [49,50,51]. In addition, we observe a significant decrease in the peak intensity for Raman peaks at 198–202 and 325–328 cm^−1^ when the concentration of the Ni acetate in the shell increases. This finding is explained by the phase transfer of the Fe_3_O_4_ to spinel form due to the higher concentration of Ni acetate (Figure 7, curves 3–5).

Analysis of the Raman spectra confirmed phase transitions of Fe_2_O_3_ to Fe_3_O_4_ and Fe_3_O_4_ to NiFe_2_O_4_ and FeNi_2_O_4_ in the core of the fibers. XRD, TEM/EDS, and Raman measurements agree with phase transitions in the core iron oxide and shell nickel oxide during nanofiber fabrication.

XPS results are presented in Figure 7b–d. Deconvolution of the Fe 2p1/2 and Fe 2p3/2 showed the presence of Fe^3+^ and Fe^2+^ oxidation states in exhibiting peaks with binding energies 725 eV (Fe^3+^)/723.9 (Fe^2+^) and 712 eV (Fe^3+^)/710(Fe^2+^), respectively [54]. Deconvolution of the Ni 2p1/2 and Ni 2p3/2 showed the presence of Ni^3+^ and Ni^2+^ oxidation states exhibiting peaks with binding energies of 873.7 eV (Ni^3+^)/872.1 eV (Fe^2+^) and 856.1 eV (Fe^3+^)/854.6 eV (Fe^2+^), respectively [55,56]. Deconvolution of O 1s peaks showed characteristic peaks at 530–529 eV, 531–532 eV, and 532.2–534 eV, corresponding to Ni-O, defect states, and adsorbed surface oxygen/water, respectively [55].

The presence of two oxidation stages, Ni^3+^/Ni^2+^ and Fe^3+^/Fe^2+^, explains phase transitions and defect formation in core–shell metal oxide nanofibers [55,57]. Particularly, for NiO, the XPS ratio of [Ni^3+^/Ni^2+^] is denoted as a key parameter for the formation of defects in NiO structure and spinel phases [57]. Table 3 summarizes the calculated ratio of XPS peaks for Ni^3+^/Ni^2+^, Fe^3+^/Fe^2+^, and oxygen peaks to evaluate the spinel phases and defect presence in the core–shell nanofibers.

Based on the obtained results, the spinel phase concentration of the defects in the NiO layer increased with the concentration of the shell precursor and achieved the highest value for samples, denoted as FeNi33 [57,58]. The Fe^3+^/Fe^2+^ ratio increased from sample Fe31 to F35. This finding matches a higher possibility of the formation of NiFe_2_O_4_ rather than of FeNi_2_O_4_ when the concentration of Ni precursor increased.

The ratio (O 1s 530 eV)/(O 1s 531 eV) shows part of the stoichiometric metal oxide. This ratio is a parameter defining the formation of defects. The reduced values of the ratio (O 1s 530 eV)/(O 1s 531eV) for NiO shell confirm our assumption about the intense formation of the defects in NiO [57,58]. Formation of defects in the shell layer could be useful for optical absorption and photocatalytic properties [58].

**Figure 8:**
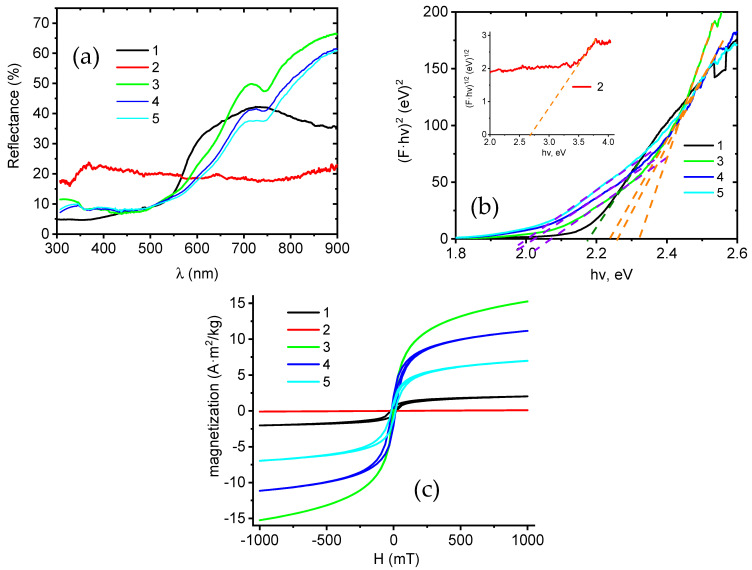
Characterization of the optical and magnetic properties of core–shell nanofibers: (**a**) diffuse reflectance spectra: 1—FeNF, 2—NiNT, 3—FeNi31, 4—FeNi33, 5—FeNi35; (**b**) band gap calculations: 1—FeNF, 2—NiNT, 3—FeNi31, 4—FeNi33, 5—FeNi35; (**c**) magnetic properties: 1—FeNF, 2—NiNT, 3—FeNi31, 4—FeNi33, 5—FeNi35.

Optical properties of the core–shell nanofibers were investigated by diffuse reflectance spectroscopy (Figure 8a). The core–shell nanofibers had a wide absorption range from 550 to 740 nm with two characteristic slopes. The measured diffuse reflectance *R* was recalculated to the Kubelka–Munk coefficient *F* as follows [59]:

(1) $$F=\frac{{\left(1-R\right)}^{2}}{2\cdot R}$$

The band gap *E_g_* of the control sample Fe_3_O_4_ and Fe_3_O_4_/NiO core–shell nanofibers was calculated using the Tauc plot for direct optical transitions [60]:

(2) $${\left(F\cdot hv\right)}^{2}=A\cdot \left(hv-E_{g}\right)$$

where *A* and *hv* are the constant and the photon energy, respectively. For NiO control samples, the power coefficient in Equation (2) changed to 0.5. We want to emphasize that the Tauc plot with a power coefficient of 0.5 was applicable only for NiO (Figure 8, curve 2). The iron oxide nanofibers and core–shell nanofibers showed direct optical transitions (Figure 8, 1, 3–5). The band gap values were calculated from linear slopes in the figure, and the following band gap values were calculated: 2.18 eV (Fe_2_O_3_), 2.76 eV (NiO), 2.08 eV and 2.33 eV (FeNi31), 2.02 eV and 2.26 eV (FeNi33), 2.02 eV and 2.23 eV (FeNi35). Fe_2_O_3_ nanostructures have a direct band gap in the range of 2–2.5 eV [61,62]. The Fe_3_O_4_ band gap energy is in the range of 2–2.2 eV [63]. NiO has a high band gap energy (2.8–4.5 eV) depending on the way of preparation, crystallinity, and defect concentration [58,64,65,66]. Spinel NiFe_2_O_4_ and FeNi_2_O_4_ have direct band gaps of 1.8–2 eV and 1.5 eV, respectively [67,68]. Therefore, the observed energies of 2.02–2.08 eV could be associated with NiFe_2_O_4_.

It was shown that defects in NiO reduced the band gap [58,69,70]. The core–shell nanofibers showed two band gaps with average values of 2.06 eV and 2.26 eV, corresponding to Fe_3_O_4_ and NiFe_2_O_4_, respectively. No band gaps in the UV region, related to the absorption of the NiO, were observed. Insignificant changes in the band gaps for Fe_3_O_4_ and NiFe_2_O_4_ with an increase in the Ni acetate concentration could be explained by size effects due to changes in the core and shell dimensions. The fabricated core–shell nanofibers absorb more light compared to bare Fe_2_O_3_. Their absorption spectra cover a higher spectral interval in the visible range, which opens perspectives in photocatalytic applications.

Samples FeNi31, FeNi33, and FeNi35 display a considerable response to the magnetic field. Magnetization curves of the samples, as well as Fe_2_O_3_ nanofibers and NiO nanotubes, are provided in Figure 8c. The magnetic properties of the samples are dominated by the Fe_3_O_4_ and NiFe_2_O_4_ content, as demonstrated by a comparison of the NiO nanotube and pure iron oxide nanofiber curves. The samples are ferromagnetic, with a pronounced hysteresis. Saturation magnetization, coercivity, and mass magnetic susceptibility of the samples are shown in Table 4.

Saturation magnetization per mass correlates inversely with Ni acetate concentration—samples with a lower Ni acetate concentration exhibit a higher M_S_. This would be expected and affirms the role of the iron oxide core in the resulting magnetic properties. Mass magnetic susceptibility follows the same trend. The magnetic coercivity of the samples is just above that expected for soft magnetic materials, and in the range corresponding to semi-hard magnetic materials.

## 4. Conclusions

A novel methodology for the fabrication of metal oxide core–shell nanofibers with advanced optical and magnetic properties has been developed. Variation of the chemical composition of precursors for core and shell and investigation of spinel formation showed that within a number of precursors (Mn, Mo, Co, and Ni), the core–shell nanostructures were formed only in the combination of Fe/Ni. Optimization of the fabrication parameters showed that the concentration of iron nitrate in the core solution plays a crucial role. After setting the optimized fabrication parameters, novel Fe_3_O_4_-Fe_2_NiO_4_/NiO core–shell nanofibers were synthesized by the co-axial electrospinning method. The TEM-EDS and XRD data confirms fabrication of Fe_3_O_4_, FeNi_2_O_4_, and Fe_2_NiO_4_ in the core and NiO in the shell. The phase transitions of Fe_2_O_3_→Fe_3_O_4_ and Fe_3_O_4_→Fe_2_NiO_4_ and the formation of nanolayers of NiO with high defect concentrations were validated by Raman, XPS, and optical spectroscopy. The high ratios of [Ni^3+^/Ni^2+^] and [Fe^3+^/Fe^2+^] states were obtained, confirming the formation of iron oxide, Fe_2_NiO_4_ spinel, and defects in the nickel oxide layer. The core–shell nanostructures showed high absorbance in the visible range. The core–shell samples showed higher magnetization compared to bare iron oxide nanofibers. Showing advanced structure, optical, and magnetic properties, the developed novel Fe_3_O_4_-Fe_2_NiO_4_/NiO core–shell nanofibers have good prospects for photocatalytic applications in the visible spectral range.

## Figures and Tables

**Table 1:** Current applications of core–shell nanofibers.

Core–Shell Nanostructure	Application	Reference
SnO_2_/TiO_2_ nanofibers	Rhodamine B photocatalytic degradation	[13]
SnO_2_-WO_3_ nanofibers	Ethanol, toluene, acetone resistive sensors	[14]
Co_3_O_4_-ZnO nanofibers	Formaldehyde resistive sensor	[15]
In_2_O_3_−SnO_2_ nanofibers	Trimethylamine resistive sensor	[16]
Fe_3_O_4_@SiO_2_ nanofibers	pH sensor, oil–water separation	[17]
Ni@NiO/NiCO_3_	Photocatalytic water splitting	[18]
Fe_3_O_4_/ZnO heterostructures	Rhodamine B photocatalytic degradation	[19]
Fe_3_O_4_@C nanofibers	Microwave absorption	[20]
Co_3_O_4_/TiO_2_ nanofibers	Acetone resistive sensor	[8]
Nylon–ZnO nanofibers	Photocatalytic degradation of Rhodamine B	[21]

**Table 2:** Dimensions of the core diameter and the shell thickness of the core–shell nanofibers.

	Core	Shell
FeNi31	174 ± 15 nm	13 ± 7 nm
FeNi33	172 ± 12 nm	23 ± 12 nm
FeNi35	178 ± 22 nm	36 ± 15 nm

**Table 3:** Summary of the XPS peak area ratios.

Sample	Ni3+ 2p3/2/Ni2+ 2p3/2	Fe3+ 2p3/2/Fe2+ 2p3/2	(O 1s 530 eV)/ (O 1s 531 eV)
FeNi31	3.2008	3.026882	0.37
FeNi33	3.887043	2.988601	0.21
FeNi35	3.667256	5.988984	0.205

**Table 4:** Magnetic properties of the samples.

Sample	M_S_* (A m^2^/kg)	H_C_ (kA/m)	H_C_ (Oe)	χ (m^3^/kg)	χ (emu/(g·kOe))
FeNi31	15.3	2.1	26.0	1.2	14.9
FeNi33	11.2	12.1	152.1	0.8	10.1
FeNi35	6.1	4.3	53.4	0.4	5.8

M_S_* denotes magnetization values recorded at H = 1 T field, standing in for saturation magnetization. H_C_ is magnetic coercivity, and χ is mass magnetic susceptibility. H_C_ and χ are expressed in both SI units and CGS notation—the duality is not required for M_S_ as the numerical values in both systems coincide.

## Data Availability

Data are contained within the article and Supplementary Materials.

## Supplementary Materials

The following supporting information can be downloaded at: https://www.mdpi.com/article/10.3390/nano15131026/s1, Figure S1. SEM images of FeCo35 samples.

## References

[1] Liu Y., Chen X., Lin X., Yan J., Yu D.G., Liu P., Yang H. (2024). Electrospun Multi-Chamber Core–Shell Nanofibers and Their Controlled Release Behaviors: A Review. Wiley Interdiscip. Rev. Nanomed. Nanobiotechnol..

[2] Lee Y., Jung S., Yun J.S. (2024). Electrospinning and Partial Etching Behaviors of Core–Shell Nanofibers Directly Electrospun on Mesh Substrates for Application in a Cover-Free Compact Air Filter. Nanomaterials.

[3] Negahdari N., Alizadeh S., Majidi J., Saeed M., Ghadimi T., Tahermanesh K., Arabsorkhi-Mishabi A., Pezeshki-Modaress M. (2024). Heat-Treated Alginate-Polycaprolactone Core-Shell Nanofibers by Emulsion Electrospinning Process for Biomedical Applications. Int. J. Biol. Macromol..

[4] Nair K.G., Vishnuraj R., Pullithadathil B. (2022). Integrated Co-Axial Electrospinning for a Single-Step Production of 1D Aligned Bimetallic Carbon Fibers@AuNPs–PtNPs/NiNPs–PtNPs towards H_2_ Detection. Mater. Adv..

[5] Sukumar T., Kadirvelu K. (2022). Core-Shell Nanofibers With Fire Retardant Properties Prepared By A Co-Axial Electrospinning Technique. ChemistrySelect.

[6] Zhang X., Aravindan V., Kumar P.S., Liu H., Sundaramurthy J., Ramakrishna S., Madhavi S. (2013). Synthesis of TiO_2_ Hollow Nanofibers by Co-Axial Electrospinning and Its Superior Lithium Storage Capability in Full-Cell Assembly with Olivine Phosphate. Nanoscale.

[7] Sahoo S.K., Panigrahi G.K., Dhal J.P., Sahoo J.K., Behera A.K., Panda P.C., Patel P., Mund S.K., Muduli S.M., Panda L. (2022). Co-Axial Electrospun Hollow MgO Nanofibers for Efficient Removal of Fluoride Ions from Water. Colloids Surf. A Physicochem. Eng. Asp..

[8] Fu L., Xu J., Liu Q., Liu C., Fan S., Ramakrishna S., Tang W. (2024). Gas Sensors Based on Co_3_O_4_/TiO_2_ Core-Shell Nanofibers Prepared by Coaxial Electrospinning for Breath Marker Acetone Detection. Ceram. Int..

[9] Rafieipour H., Vaezi M.R., Kazemzadeh A. (2016). Synthesis and Characterisation of Ceramic Core/Shell Nanofibres via Single Stage Co-Axial Electrospinning. Micro Nano Lett..

[10] Zhou Y., Li Y., Hou Y., Wang C., Yang Y., Shang J., Cheng X. (2023). Core-Shell Catalysts for the Elimination of Organic Contaminants in Aqueous Solution: A Review. Chem. Eng. J..

[11] Wang I., Liu L., Yu S., Lai N.C., Gao Y., Li Z., Liu J., Wang W. (2024). Highly Sintering-Resistant Iron Oxide with a Hetero-Oxide Shell for Chemical Looping Water Splitting. Int. J. Hydrog. Energy.

[12] Sethy P.P., Pani T.K., Rout S., Sundaray B. (2023). Structural and Magnetic Properties of Ni/C Core–Shell Nanofibers Prepared by One Step Co-Axial Electrospinning Method. J. Mater. Sci. Mater. Electron..

[13] Peng X., Santulli A.C., Sutter E., Wong S.S. (2012). Fabrication and Enhanced Photocatalytic Activity of Inorganic Core–Shell Nanofibers Produced by Coaxial Electrospinning. Chem. Sci..

[14] Li F., Gao X., Wang R., Zhang T. (2018). Design of WO_3_-SnO_2_ Core-Shell Nanofibers and Their Enhanced Gas Sensing Performance Based on Different Work Function. Appl. Surf. Sci..

[15] Gao X., Li F., Wang R., Zhang T. (2018). A Formaldehyde Sensor: Significant Role of p-n Heterojunction in Gas-Sensitive Core-Shell Nanofibers. Sens. Actuators B Chem..

[16] Li F., Gao X., Wang R., Zhang T., Lu G., Barsan N. (2016). Design of Core-Shell Heterostructure Nanofibers with Different Work Function and Their Sensing Properties to Trimethylamine. ACS Appl. Mater. Interfaces.

[17] Chen Q., Liu J., Tang L., Zeng Z., Zhu B. (2024). A Novel Ex-Situ Method to Fabricate PH-Responsive Material Based on Core-Shell Fe_3_O_4_@SiO_2_ Nanoparticles for Multi-Functional Oil-Water Separation and Efficient Recycling. J. Env. Chem. Eng..

[18] Talebi P., Singh H., Rani E., Huttula M., Cao W. (2021). Surface Plasmon-Driven Photocatalytic Activity of Ni@NiO/NiCO_3_ Core–Shell Nanostructures. RSC Adv..

[19] Pham H.L., Nguyen V.D., Nguyen V.K., Le T.H.P., Ta N.B., Pham D.C., Tran Q.T., Dang V.T. (2021). Rational Design of Magnetically Separable Core/Shell Fe_3_O_4_/ZnO Heterostructures for Enhanced Visible-Light Photodegradation Performance. RSC Adv..

[20] Zhang T., Huang D., Yang Y., Kang F., Gu J. (2013). Fe_3_O_4_/Carbon Composite Nanofiber Absorber with Enhanced Microwave Absorption Performance. Mater. Sci. Eng. B.

[21] Kayaci F., Ozgit-Akgun C., Donmez I., Biyikli N., Uyar T. (2012). Polymer-Inorganic Core-Shell Nanofibers by Electrospinning and Atomic Layer Deposition: Flexible Nylon-ZnO Core-Shell Nanofiber Mats and Their Photocatalytic Activity. ACS Appl. Mater. Interfaces.

[22] Lys A., Zabolotnii V., Čaplovičová M., Tepliakova I., Berzins A., Sahul M., Čaplovič Ľ., Pogrebnjak A., Iatsunskyi I., Viter R. (2024). Core-Shell Nanofibers of ZnFe_2_O_4_/ZnO for Enhanced Visible-Light Photoelectrochemical Performance. J. Alloys Compd..

[23] Okpara E.C., Olatunde O.C., Wojuola O.B., Onwudiwe D.C. (2023). Applications of Transition Metal Oxides and Chalcogenides and Their Composites in Water Treatment: A Review. Environ. Adv..

[24] Krishnan A., Swarnalal A., Das D., Krishnan M., Saji V.S., Shibli S.M.A. (2024). A Review on Transition Metal Oxides Based Photocatalysts for Degradation of Synthetic Organic Pollutants. J. Environ. Sci..

[25] Wang W., Zhou Y.H., Sun P.K., Liu L.G., Guo C., Wang X.L., Zhang T.F., Cong Y.N., Wei Z.B. (2024). One-Step Synthesis of Double-Walled NiCo_2_O_4_ Hollow Nanotubes Using Hollow Fiber Templates Created by Airflow-Coaxial Electrospinning. Vacuum.

[26] Sethy P.P., Sundaray B. (2023). Magnetic Behavior of Fe_3_O_4_@C Nanofibers by a Facile Co-Axial Electrospinning Method. Nanotechnology.

[27] Yoon J., Yang H.S., Lee B.S., Yu W.R. (2018). Recent Progress in Coaxial Electrospinning: New Parameters, Various Structures, and Wide Applications. Adv. Mater..

[28] Li M., Zheng Y., Xin B., Xu Y. (2020). Coaxial Electrospinning: Jet Motion, Core-Shell Fiber Morphology, and Structure as a Function of Material Parameters. Ind. Eng. Chem. Res..

[29] Sun B., Duan B., Yuan X. (2006). Preparation of Core/Shell PVP/PLA Ultrafine Fibers by Coaxial Electrospinning. J. Appl. Polym. Sci..

[30] Hieu N.T., Baik S.J., Jun Y., Lee M., Chung O.H., Park J.S. (2014). Electrospun Coaxial Titanium Dioxide/Carbon Nanofibers for Use in Anodes of Dye-Sensitized Solar Cells. Electrochim. Acta.

[31] Li M., Xu W., Wang W., Liu Y., Cui B., Guo X. (2014). Facile Synthesis of Specific FeMnO_3_ Hollow Sphere/Graphene Composites and Their Superior Electrochemical Energy Storage Performances for Supercapacitor. J. Power Sources.

[32] Moura J.V.B., Pinheiro G.S., Freire P.T.C., Filho J.M., Saraiva G.D., Viana B.C., Luz-Lima C. (2016). High-Pressure Raman Scattering on Fe_2_(MoO_4_)_3_ Microcrystals Obtained by a Hydrothermal Method. Vib. Spectrosc..

[33] Yao J., Zhou Y., Yan J.M., Jiang Q. (2021). Regulating Fe_2_(MoO_4_)_3_ by Au Nanoparticles for Efficient N_2_ Electroreduction under Ambient Conditions. Adv. Energy Mater..

[34] Khanam S., Zakaria A.K.M., Ahsan M.H., Datta T.K., Aktar S., Liba S.I., Hossain S., Das A.K., Kamal I., Yunus S.M. (2015). Study of the Crystallographic and Magnetic Structure in the Nickel Substituted Cobalt Ferrites by Neutron Diffraction. Mater. Sci. Appl..

[35] El-Masry M.M., Ramadan R. (2022). The Effect of CoFe_2_O_4_, CuFe_2_O_4_ and Cu/CoFe_2_O_4_ Nanoparticles on the Optical Properties and Piezoelectric Response of the PVDF Polymer. Appl. Phys. A Mater. Sci. Process.

[36] Meng L., Dong J., Chen J., Li L., Huang Q., Lu J. (2023). Activation of Peracetic Acid by Spinel FeCo_2_O_4_ Nanoparticles for the Degradation of Sulfamethoxazole. Chem. Eng. J..

[37] Bertolucci E., Galletti A.M.R., Antonetti C., Marracci M., Tellini B., Piccinelli F., Visone C. Chemical and Magnetic Properties Characterization of Magnetic Nanoparticles. Proceedings of the 2015 IEEE Instrumentation and Measurement Technology Conference.

[38] Yan H., Zhang D., Xu J., Lu Y., Liu Y., Qiu K., Zhang Y., Luo Y. (2014). Solution Growth of NiO Nanosheets Supported on Ni Foam as High-Performance Electrodes for Supercapacitors. Nanoscale Res. Lett..

[39] Ma L., Shi X., Zhang X., Li L. (2019). Electrospinning of Polycaprolacton/Chitosan Core-Shell Nanofibers by a Stable Emulsion System. Colloids Surf. A Physicochem. Eng. Asp..

[40] Wang L., Yang H., Hou J., Zhang W., Xiang C., Li L. (2017). Effect of the Electrical Conductivity of Core Solutions on the Morphology and Structure of Core–Shell CA-PCL/CS Nanofibers. New J. Chem..

[41] Qayoom M., Shah K.A., Pandit A.H., Firdous A., Dar G.N. (2020). Dielectric and Electrical Studies on Iron Oxide (α-Fe_2_O_3_) Nanoparticles Synthesized by Modified Solution Combustion Reaction for Microwave Applications. J. Electroceram..

[42] Aytan E., Debnath B., Kargar F., Barlas Y., Lacerda M.M., Li J.X., Lake R.K., Shi J., Balandin A.A. (2017). Spin-Phonon Coupling in Antiferromagnetic Nickel Oxide. Appl. Phys. Lett..

[43] Jaiswal R., Ranganath K.V.S. (2021). Carbon Nanoparticles on Magnetite: A New Heterogeneous Catalyst for the Oxidation of 5-Hydroxymethylfurfural (5-HMF) to 2,5-Diformoylfuran (DFF). J. Inorg. Organomet. Polym. Mater..

[44] Hai N.H., Phu N.D., Luong N.H., Chau N., Chinh H.D., Hoang L.H., Leslie-Pelecky D.L. (2008). Mechanism for Sustainable Magnetic Nanoparticles under Ambient Conditions. J. Korean Phys. Soc..

[45] Baghaie Yazdi M., Choi K.Y., Wulferding D., Lemmens P., Alff L. (2013). Raman Study of the Verwey Transition in Magnetite Thin Films. New J. Phys..

[46] Gebretinsae H.G., Tsegay M.G., Nuru Z.Y. (2021). Biosynthesis of Nickel Oxide (NiO) Nanoparticles from Cactus Plant Extract. Mater. Today Proc..

[47] Caso D., Serrano A., Jaafar M., Prieto P., Kamra A., González-Ruano C., Aliev F.G. (2024). Microwave Field-Induced Changes in Raman Modes and Magnetic Force Images of Antiferromagnetic NiO Films. Condens. Matter.

[48] Mironova-Ulmane N., Kuzmin A., Sildos I., Puust L., Grabis J. (2019). Magnon and Phonon Excitations in Nanosized NiO. Latv. J. Phys. Tech. Sci..

[49] Alhashem Z., Awada C., Ahmed F., Farha A.H. (2021). Structural and Magnetic Properties Study of Fe_2_O_3_/NiO/Ni_2_FeO_4_ Nanocomposites. Crystals.

[50] Sedrati C., Alleg S., Boussafel H., Bendali Hacine A. (2021). Structure and Magnetic Properties of Nickel Ferrites Synthesized by a Facile Co-Precipitation Method: Effect of the Fe/Ni Ratio. J. Mater. Sci. Mater. Electron..

[51] Soam A., Kumar R., Thatoi D., Singh M. (2020). Electrochemical Performance and Working Voltage Optimization of Nickel Ferrite/Graphene Composite Based Supercapacitor. J. Inorg. Organomet. Polym. Mater..

[52] Liu D., Li D., Yang D. (2017). Size-Dependent Magnetic Properties of Branchlike Nickel Oxide Nanocrystals. AIP Adv..

[53] Sahu B., Panigrahi U.K., Chakravarty S., Hussain S., Mallick P. (2023). Structural, Optical, and Magnetic Properties of NiO/NiFe_2_O_4_ Nanocomposites. Appl. Phys. A Mater. Sci. Process.

[54] Ai Q., Yuan Z., Huang R., Yang C., Jiang G., Xiong J., Huang Z., Yuan S. (2019). One-Pot Co-Precipitation Synthesis of Fe_3_O_4_ Nanoparticles Embedded in 3D Carbonaceous Matrix as Anode for Lithium Ion Batteries. J. Mater. Sci..

[55] Imran M., Coskun H., Khan N.A., Ouyang J. (2021). Role of Annealing Temperature of Nickel Oxide (NiOx) as Hole Transport Layer in Work Function Alignment with Perovskite. Appl. Phys. A Mater. Sci. Process.

[56] Huang W., Ding S., Chen Y., Hao W., Lai X., Peng J., Tu J., Cao Y., Li X. (2017). 3D NiO Hollow Sphere/Reduced Graphene Oxide Composite for High-Performance Glucose Biosensor. Sci. Rep..

[57] Yu Y., Pan M., Zhang Z., An Z., Wang Y., Hu X. (2023). Improved Electrocatalytic Methanol Oxidation of NiO Nanosheet Arrays with Synergistic High Surface Oxygen Vacancy and Ni^3+^/Ni^2+^ Ratio. Int. J. Hydrog. Energy.

[58] Jouini K., Raouafi A., Dridi W., Daoudi M., Mustapha B., Chtourou R., Hosni F. (2019). Investigation of Gamma-Ray Irradiation Induced Phase Change from NiO to Ni_2_O_3_ for Enhancing Photocatalytic Performance. Optik.

[59] Kawrani S., Boulos M., Bekheet M.F., Viter R., Nada A.A., Riedel W., Roualdes S., Cornu D., Bechelany M. (2020). Segregation of Copper Oxide on Calcium Copper Titanate Surface Induced by Graphene Oxide for Water Splitting Applications. Appl. Surf. Sci..

[60] Choudhary S., Hasina D., Saini M., Ranjan M., Mohapatra S. (2022). Facile Synthesis, Morphological, Structural, Photocatalytic and Optical Properties of ZnFe_2_O_4_-ZnO Hybrid Nanostructures. J. Alloys Compd..

[61] Tahir D., Ilyas S., Rahmat R., Heryanto H., Fahri A.N., Rahmi M.H., Abdullah B., Hong C.C., Kang H.J. (2021). Enhanced Visible-Light Absorption of Fe_2_O_3_ Covered by Activated Carbon for Multifunctional Purposes: Tuning the Structural, Electronic, Optical, and Magnetic Properties. ACS Omega.

[62] Leão-Neto V.S., da Silva A.C., Camargo L.P., Da Silva Pelissari M.R., da Silva P.R.C., Parreira P.S., Segatelli M.G., Dall′Antonia L.H. (2020). Fabrication of RGO/α-Fe_2_O_3_ Electrodes: Characterization and Use in Photoelectrocatalysis. J. Mater. Sci. Mater. Electron..

[63] Delice S., Isik M., Gasanly N.M. (2024). Temperature-Dependent Tuning of Band Gap of Fe_3_O_4_ Nanoparticles for Optoelectronic Applications. Chem. Phys. Lett..

[64] Haider A.J., Al-Anbari R., Sami H.M., Haider M.J. (2019). Enhance Preparation and Characterization of Nickel-Oxide as Self-Cleaning Surfaces. Energy Procedia.

[65] Hashem M., Saion E., Al-Hada N.M., Kamari H.M., Shaari A.H., Talib Z.A., Paiman S.B., Kamarudeen M.A. (2016). Fabrication and Characterization of Semiconductor Nickel Oxide (NiO) Nanoparticles Manufactured Using a Facile Thermal Treatment. Results Phys..

[66] Hosny N.M. (2011). Synthesis, Characterization and Optical Band Gap of NiO Nanoparticles Derived from Anthranilic Acid Precursors via a Thermal Decomposition Route. Polyhedron.

[67] Zhang Y., Qu H., Gang C., Guan H., Dong C., Yin Z. (2023). Porous, Tremella-like NiFe_2_O_4_ with Ultrathin Nanosheets for Ppb-Level Toluene Detection. Crystals.

[68] Tong S.K., Chi P.W., Kung S.H., Wei D.H. (2018). Tuning Bandgap and Surface Wettability of NiFe_2_O_4_ Driven by Phase Transition. Sci. Rep..

[69] Bhanuchandar S., Vinothkumar G., Arunkumar P., Sribalaji M., Keshri A.K., Babu K.S. (2023). Unravelling the Role of Cationic Ni^2+^ Vacancies and Ni^3+^ Ions in Non-Stoichiometric NiO: Breakdown of Anti-Ferromagnetic Ordering and Large Exchange Bias. J. Mater. Sci..

[70] Barwant M., Karande V., Basnet P., Kumar D., Sargazi S., Mirzaei M., Jabir M.S., Sanap D., Ghotekar S. (2024). Insights into the Antioxidant and Anticancer Properties of Novel Biologically Synthesized NiO/Ni_2_O_3_ Nanoparticles Using Sargassum Tenerrimum. J. Solgel Sci. Technol..

