# Supplementary material for: Novel Core–Shell Metal Oxide Nanofibers with Advanced Optical and Magnetic Properties Deposited by Co-Axial Electrospinning
Source: Nanomaterials (Basel). 2025 Jul 2;15(13):1026. doi: 10.3390/nano15131026 (PMC12250830; doi:10.3390/nano15131026)
Supplement: Supplementary file 1 [file nanomaterials-15-01026-s001.zip › nanomaterials-3705336-supplementary.pdf]

nanomaterials

Novel Core–Shell Metal Oxide Nanofibers with Advanced Optical and Magnetic Properties Deposited by Co-Axial Electrospinning

Roman Viter [1,*], Viktor Zabolotnii [1], Martin Sahul [2], Mária Čaplovičová [3], Iryna Tepliakova [1], Viesturs Sints [1] and Ambra Fioravanti [4]

Supplementary information

SEM images of FeCo nanostructures, obtained by co-axial electrospinning are shown in Figure S1. The obtained samples mainly showed no nanofibers. Optimization of parameters didn't improve the shape of the final nanostructures.

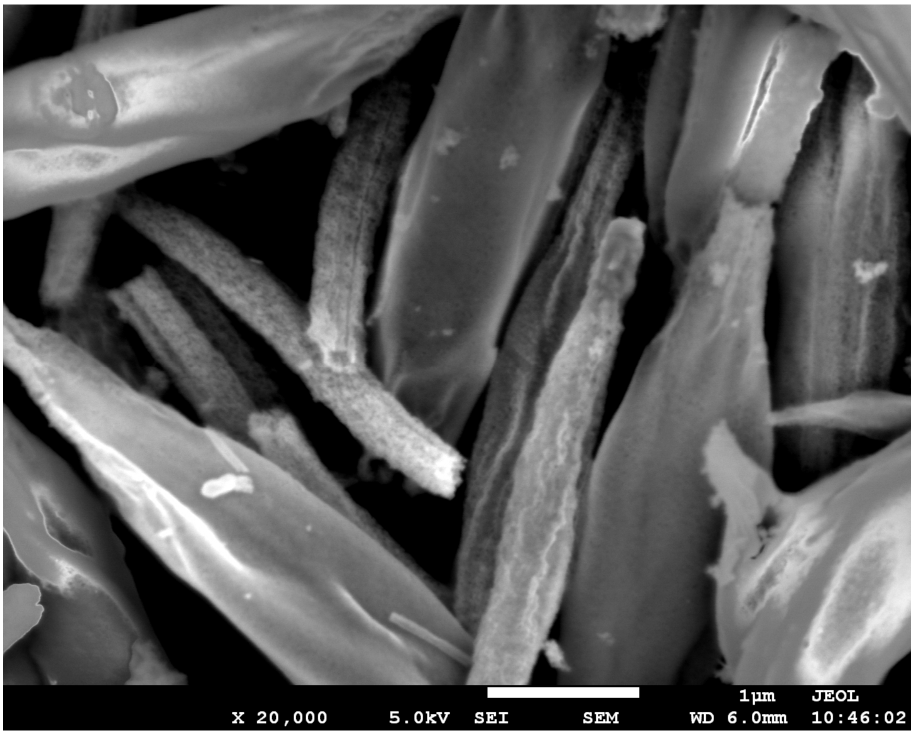

Figure S1. SEM images of FeCo35 samples.

https://doi.org/10.3390/nano15131026
